# HIV Disease Progression Among Antiretroviral Therapy Patients in Zimbabwe: A Multistate Markov Model

**DOI:** 10.3389/fpubh.2019.00326

**Published:** 2019-11-15

**Authors:** Zvifadzo Matsena Zingoni, Tobias F. Chirwa, Jim Todd, Eustasius Musenge

**Affiliations:** ^1^Division of Epidemiology and Biostatistics, School of Public Health, Faculty of Health Sciences, University of the Witwatersrand, Johannesburg, South Africa; ^2^Ministry of Health and Child Care, National Institute of Health Research, Harare, Zimbabwe; ^3^Department of Population Health, London School of Hygiene and Tropical Medicine, London, United Kingdom

**Keywords:** antiretroviral therapy, disease progression, human immunodeficiency virus, mortality, multistate Markov models, Zimbabwe

## Abstract

**Background:** Antiretroviral therapy (ART) impact has prolonged survival of people living with HIV. We evaluated HIV disease progression among ART patients using routinely collected patient-level data between 2004 and 2017 in Zimbabwe.

**Methods:** We partitioned HIV disease progression into four transient CD4 cell counts states: state 1 (CD4 ≥ 500 cells/μl), state 2 (350 cells/μl ≤ CD4 < 500 cells/μl), state 3 (200 cells/μl ≤ CD4 < 350 cells/μl), state 4 (CD4 < 200 cells/μl), and the absorbing state death (state 5). We proposed a semiparametric time-homogenous multistate Markov model to estimate bidirectional transition rates. Covariate effects (age, gender, ART initiation period, and health facility level) on the transition rates were assessed.

**Results:** We analyzed 204,289 clinic visits by 63,422 patients. There were 24,325 (38.4%) patients in state 4 (CD4 < 200) at ART initiation, and 7,995 (12.6%) deaths occurred by December 2017. The overall mortality rate was 3.9 per 100 person-years. The highest mortality rate of 5.7 per 100 person-years (4,541 deaths) was from state 4 (CD4 < 200) compared to other states. Mortality rates decreased with increase in time since ART initiation. Health facility type was the strongest predictor for immune recovery. Provincial or central hospital patients showed a diminishing dose–response effect on immune recovery by state from a hazard ratio (HR) of 8.30 [95% confidence interval (95% CI), 6.64–10.36] (state 4 to 3) to HR of 3.12 (95% CI, 2.54–4.36) (state 2 to 1) compared to primary healthcare facilities. Immune system for male patients was more likely to deteriorate, and they had a 32% increased mortality risk (HR, 1.32; 95% CI, 1.23–1.42) compared to female patients. Elderly patients (45+ years) were more likely to immune deteriorate compared to 25–34 years age group: HR, 1.35; 95% CI, 1.18–1.54; HR, 1.56; 95% CI, 1.34–1.81 and HR, 1.53; 95% CI, 1.32–1.79 for states 1 to 2, state 2 to 3, and states 3 to 4, respectively.

**Conclusion:** Immune recovery was pronounced among provincial or central hospitals. Male patients with lower CD4 cell counts were at a higher risk of immune deterioration and mortality, while elderly patients were more likely to immune deteriorate. Early therapeutic interventions when the immune system is relatively stable across gender and age may contain mortality and increase survival outcomes. Interventions which strengthen ART services in primary healthcare facilities are essential.

## Introduction

Over the last 15 years, remarkable strides have been made to tackle the human immunodeficiency virus (HIV) pandemic globally. The Sub-Saharan Africa (SSA) region is disproportionately affected by the pandemic, accounting for more than 50% of people living with HIV (PLHIV) (1, 2). Antiretroviral therapy (ART) treatment remains the backbone of HIV treatment and prevention. Globally, it was estimated that 59% of PLHIV were receiving ART in 2017 (2).

Zimbabwe is one of the countries in SSA affected by HIV infection. The country had an estimated 1.3 million PLHIV and an adult prevalence of 13.3% in 2017 (3). The country's ART coverage was estimated at 84% for adult patients in the same year (3). There has been a reduction in the number of new HIV infections and HIV-related deaths between 2010 and 2016 (4), and this can be attributed to ART as the main driver. ART drugs help boost the immune system of the PLHIV (5), which leads to viral load suppression, and an increase in CD4 cell counts. Both CD4 cell counts and viral load are key prognostic markers in measuring HIV disease progression (6). The World Health Organization (WHO) recommends the use of viral load in monitoring HIV disease progression among ART patients. Viral load suppression has been incorporated as one of the ultimate indicators in the UNAIDS 90-90-90 fast track targets (7). However, over the years, CD4 cell counts have been extensively used as a marker for HIV disease progression.

Disease progression and immune recovery can be evaluated using either time homogenous or time inhomogenous semiparametric multistate Markov models using CD4 cell counts (8). Application of these models in the assessment of HIV progression has been used in the past decades (9), and many studies have recently employed them (9–14). The use of CD4 cell counts as a prognostic marker for HIV disease progression has been well-documented (11, 12, 15–17). However, across studies, there is variation in terms of the number of discrete multistate model states, the cutoff points defining each state, the type of transitions which can either be reversible or irreversible, and the number of transitions to be estimated.

In this new era of “test and treat all” regardless of CD4 cell counts, HIV patients are initiated on ART as soon as they are tested positive. However, this does not rule out the possibility of having patients who present late for HIV diagnosis with an advanced immune deterioration. This put forward the importance of understanding the HIV disease progression across all possible disease states since patients are initiated on ART with different immune stages. Once the HIV-infected patients are initiated on ART, they are still exposed to difference factors which may still affect their ART adherences. Therefore, it is important to understand the different trajectories that patients follow in HIV disease progression to inform policy makers on possible interventions to be carried out and encourage the patients on the need to adhere on ART for their own improved health outcomes, all in the quest to achieve zero HIV incidences by 2030 (18).

Zimbabwe adopted the WHO recommendation on the decentralization of ART services from higher levels of care to primary healthcare (PHC) facilities to increase ART coverage, access and uptake to those in need, and increase ART patient retention. This approach resulted in lessening the work burden in the higher levels of care (19) through task shifting of HIV management and ART service cascading down to PHC facilities (20–23). As a result, the ART sites in Zimbabwe increased from 282 in 2008 to 1,556 in 2017 (3). However, in primary health care, patient turnaround time is increased, there is lack of resources and skilled personnel, which may compromise the quality of service delivery; consequently, ART outcomes are compromised. Therefore, there is a gap to understand HIV progression patterns among ART patients after ART decentralization since the health facility type that a patient is enrolled in may influence their progression or recovery patterns.

This study aims to describe HIV disease progression and immune recovery implementing the multistate model approach based on CD4 cell counts intermediate states among adult patients on ART in Zimbabwe using patient-level data adjusting for the health facility type. The multistate model provides an in-depth understanding on the general immune deterioration (decrease in CD4 cell count) patterns, immune recovery (increase in CD4 cell count) patterns, and death outcome. Unique to this study is the inclusion of the health facility type in the analysis to account for ART services decentralization effect on transition rates.

## Materials and Methods

The study was carried out in Zimbabwe, a country with eight provinces and two metropolitans. The country is land-locked bordered by South Africa, Botswana, Mozambique, and Zambia. We conducted a retrospective analysis of cohort data from a sample of PLHIV receiving ART under the Zimbabwe national ART program. We used individual records from 538 health facilities linked to the electronic patient management system (ePMS) (3). From patients attending these health facilities, all routine clinic visits with CD4 count data were used from 1st January 2004 to 31st December 2017 in this analysis.

We included patients aged 15 years and above at ART initiation (baseline) with complete ART initiation dates, gender, and subsequent follow-up information from the dataset. We excluded patients with no information on CD4 cell counts and patients with baseline CD4 measurements only. We also excluded patients who were classified as lost to follow-up or who transferred to other health facilities to reduce the complexity of the multistate model. The patients who were alive at the end of the study were right censored at their last clinic visit before 31st December 2017.

We extracted demographic characteristics such as age (15–24, 25–34, 35–44, and 45+ years), gender, and education level (none, primary, secondary, tertiary). We also extracted data on health facility type (primary health care, district, provincial, or central hospitals) and time of ART initiation (2004–2007, 2008–2012, and 2013–2017). Clinical characteristics included for analysis were regimen type (first line, second line), WHO clinical staging (WHO I/II, WHO III/IV), tuberculosis status (negative, positive, not assessed) from the routine monitoring records of each visit to the clinic by the patients. HIV disease progression was defined using the WHO-based CD4 cell counts bands of HIV-related immunodeficiency: the no significant immunodeficiency (CD4 ≥ 500 cells/μl) as state 1, the mild immunodeficiency (350 cells/μl ≤ CD4 < 500 cells/μl) as state 2, the advanced immunodeficiency (200 cells/μl ≤ CD4 < 350 cells/μl) as state 3, the severe immunodeficiency (CD4 < 200 cells/μl) as state 4, and the absorbing state death as state 5.

### Statistical Analysis

**Figure 1:**
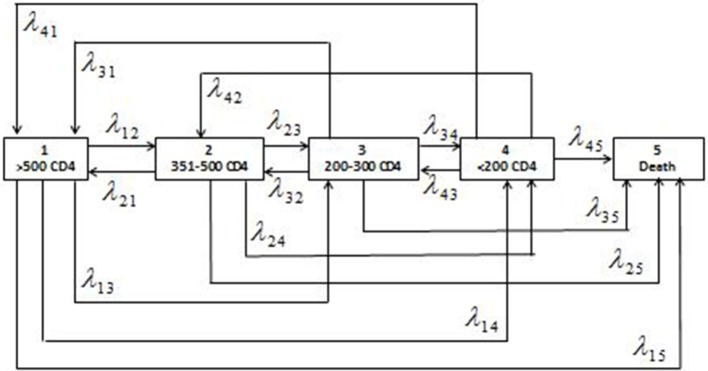
Schematic presentation of the five-staged reversible multistate Markov model with the states of HIV defined as the ranges of CD4 cell counts (cells/μl) and the corresponding individual transition intensities (λ_*jk*_where *j* = 1,2,3,4 and *k* = 1,2,3,4,5).

The patient's retrieved data were cleaned and managed in Stata 15.1 (24). All the preliminary analyses were conducted in Stata software. After data argumentation, the five-staged semiparametric time homogenous multistate Markov model was fitted in R software (25) using the *msm* package. We fitted a model with reversible transitions (26); therefore, states 1–4 were transient states, while state 5 was non-transient as depicted in Figure 1.

The fitted model was adjusted for demographic factors (sex, health facility type, and ART initiation period). The semiparametric time homogenous multistate Markov estimated transition intensities (transition rates or hazard rates), transition probabilities (survival function) between the defined CD4 cell count states, mean sojourn time, and the total length of stay in states before making any transitions. Time-varying mortality rates were estimated using time inhomogenous model, which assumes that the transitions change with time, and this reflect the reality in infectious disease progression models; hence, this is normally the preferred model. These models usually assume the Markovian process that the transition intensity depends only on the current time and state occupied, i.e., it is independent of the previous transitions. In other terms, these models were assumed to have “memory loss.” We used the *markovchain* library in R to test if the Markov assumption is satisfied. The null hypothesis of this test is that the Markov property holds. We randomly selected patients' sequences to be tested and we obtained *p* > 0.05; therefore, we failed to reject the null hypothesis that the sequences are Markovian.

### The Multistate Markov Model

A multistate process is a stochastic process [*X*(*t*), *t* ∈ *T*] with finite state space*S* = {1, 2, 3, 4, 5} where *T* = [0, τ] τ < ∞ is the period of observation (27). These models can either be discrete-time Markov chains (transitions occur at fixed points in time) or continuous-time Markov chains (transitions occur at any point in time) (28). For a continuous time Markovian process, the transition intensity (instantaneous incidence rate), λ_*jk*_(*t*), of a patient from state *X*(*t*) = *j* at time *t* to state *k* at time *t* + δ*t* is defined as:

(1) $$\begin{array}{r} \lambda_{jk}\left(t\right)=\frac{d}{dt}{p_{jk}|}_{t=0}=\underset{\delta t\to 0}{\lim}\frac{p_{jk}\left(t,t\;+\;\delta t\right)}{\delta t}\\ =\underset{\delta t\to 0}{\lim}\frac{p_{jk}\left\{X\left(t\;+\;\delta t\right)\;=\;k|X\left(t\right)\;=\;j\right\}}{\delta t} \end{array}$$

where *p*_*jk*_ is the probability from state *j* to *k*, δ*t* is the change in time. For example, in our case, the transition intensities in Equation (1) form the (*j, k*) entry of the transition rate matrix, denoted by *Q*(*t*):

$$\begin{array}{l} \begin{array}{c} Q\left(t\right)=\left(\begin{array}{ccccc} -\lambda_{1▪} & \lambda_{12} & \lambda_{13} & \lambda_{14} & \lambda_{15}\\ \lambda_{21} & -\lambda_{2▪} & \lambda_{23} & \lambda_{24} & \lambda_{25}\\ \lambda_{31} & \lambda_{32} & -\lambda_{3▪} & \lambda_{34} & \lambda_{35}\\ \lambda_{41} & \lambda_{42} & \lambda_{43} & -\lambda_{4▪} & \lambda_{45}\\ 0 & 0 & 0 & 0 & 0 \end{array}\right)\;\\ \; \end{array} \end{array}$$

whose rows sum to 0, that is$\sum_{k\in S}\lambda_{jk}=0\;for\;all\;j$, and the diagonal entries (interpreted as changes in transition probability) are defined by conversion as $\lambda_{jj}(t)=\lambda_{j▪}=-\sum_{j\neq k}\lambda_{jk}(t)\text{ for all }j\in S$. These transition intensities under the Markov process can be calculated as the product of the flow rate μ_*j*_ and the conditional probability of a transition to state*k*, given that a transition is made*j* ≠ *k*(ρ_*jk*_). From the *Q*(*t*) values, we can calculate the probability that the next state after state *j* is state*k*, for each *j* and *k* calculated as (*p*_*jk*_ = −λ_*jk*_/λ_*j*▪_). Once the transition intensity matrix is obtained, the transition probability matrix can be obtained using the Chapman–Kolmogorov forward differential equations. The detailed explanation is provided in Appendix. The probability matrix can be computed from the estimated transition intensities using *P*(*t*) = exp[*Q*(*t*)] where [*P*(*t*)] is the transition probability matrix defined as:

$$\begin{array}{l} P\left(t\right)=\exp\left[Q\left(t\right)\right]=\left(\begin{array}{ccccc} \pi_{11} & \pi_{12} & \pi_{14} & \pi_{14} & \pi_{15}\\ \pi_{21} & \pi_{22} & \pi_{23} & \pi_{24} & \pi_{25}\\ \pi_{31} & \pi_{32} & \pi_{33} & \pi_{34} & \pi_{35}\\ \pi_{41} & \pi_{42} & \pi_{43} & \pi_{44} & \pi_{45}\\ 0 & 0 & 0 & 0 & 1 \end{array}\right) \end{array}$$

The probability (π_*jk*_) that a patient in state *j* at time *t* will be in state *k* at time *t* + δ*t* is given by:

(2) $$\begin{array}{r} \pi_{jk}\left(s,t\right)=P\left[X\left(t+\delta t\right]=k|X\left(s\right)=j\right) \end{array}$$

where*s, t* ∈ *T*, and *s* ≤ *t*. These transition probabilities satisfy the following conditions:

(i)$\pi_{jk}(t+s)=\sum_{r\in S}\pi_{jr}(t)\;\pi_{rk}(s)$ for all *t* ≥ 0, *s* ≥ 0 and *j, k* ∈ *S*;

(ii)$\sum_{k\in S}\pi_{jk}(t)=1\text{ for all }j\in S\text{ and }t\geq 0$ and

(iii) π_*jk*_(*t*) ≥ 0 for all *j, k* ∈ *S* and *t* ≥ 0.

The maximum likelihood procedures (8, 29) can be used to estimate these transition intensities as a product of probabilities of transition between observed states, overall individuals *i* = 1, 2, .., *M* and observation times *r*which are observed *n* times, as shown below:

(3) $$\begin{array}{r} L\left(Q\right)=\overset{M}{\underset{i=1}{\prod }}\overset{n_{i}-1}{\underset{r=1}{\prod }}L_{i,j}=\underset{i,r}{\prod }\pi \;s\left(t_{ir}\right)s\left(t_{i,r+1}\right)\left(t_{i,r+1}-t_{ir}\right) \end{array}$$

Each component *L*_*i, r*_ is the entry of the transition probability matrix and the $s{\left(t_{ir}\right)}^{th}$ row and the $s{\left(t_{i,r+1}\right)}^{th}$ column, evaluated at a pair of consecutive observed state at times*t*_*r*_ and*t*_*r*+1_. This likelihood function, *L*(*Q*), is maximum in terms of log(λ_*jk*_)to compute the estimates ofλ_*jk*_, using the standard optimization algorithms which make use of the derivatives of the likelihood. This likelihood assumes that the sampling times are ignorable (non-informative).

### The Total Length of Stay and Mean Sojourn Time

The mean sojourn time is defined as the mean expected holding time or the average time a patient spends in each state in a single stay before making any transition to other states. The average length of stay in a single state before making any transitions to either lower or higher CD4 cell count states is estimated by a negative inverse of the *j*^*th*^ diagonal entry of *Q*(*t*), that is (−1/λ_*jj*_). The total length of stay, *L*_*k*_, in each of the four states excluding death is defined as the anticipated exposure time spent by an individual in each state during the study period before death. This time is estimated as time spent in state *k* between two successive time points (*t*1, *t*2) given by:

(4) $$\begin{array}{r} L_{k}=\int_{t1}^{t2}P_{jk}\left(t\;\right)dt \end{array}$$

where *j* is the initial state which usually is equal to one and is useful in the presence of reversible transitions.

### Semiparametric Regression Model

To adjust for the effects of the covariates on the transition rates, we proposed a semiparametric Cox proportional hazard regression model. The transition rates depend on the covariates vector matrix ***Z***, that is,

(5) $$\begin{array}{r} \lambda_{jk}\left[t|\text{Z}\left(t\right)\right]=\;\lambda_{jk0}\text{exp}\left[\beta_{jk}^{T}\text{Z}\left(t\right)\right] \end{array}$$

where $\beta_{jk}={\left(\beta_{jk1},\beta_{jk2},...,\beta_{jkz}\right)}^{T}$ is a vector of the regression coefficients associated with vector ***Z*** (*t*) for the transition from state *j* to state *k*. The baseline hazard function is denoted byλ_*jk*0_. In this study, we assumed time-independent covariates. Parameter estimation was based on the maximization of the hazard function (the transitional intensities). We fitted eight models in total [starting with a no covariates (unadjusted) model, followed by four univariate models and three with at least two covariates]. The additional covariates after the univariate models were added sequentially and only covariates without missing information were considered in the adjusted model.

### Model Diagnostics

Selection of model of best fit with covariates was performed using a likelihood ratio test define as $-2\ln(L_{s}(\overset{\wedge }{\theta })/L_{g}(\overset{\wedge }{\theta })$, where $L_{s}\left(\overset{\wedge }{\theta }\right)$ is the likelihood of the reduced (no covariate) model $L_{g}\left(\overset{\wedge }{\theta }\right)$ and is the likelihood of the full (with covariates) model, which follows a chi-square distribution with *n* degrees of freedom. Significance was set at 5% level of significance. The aim was to get a parsimonious model that explains best the model.

### Ethical Considerations

We used data with no personal identification; however, we used the individual unique identifier for the analysis. We sort permission to use the dataset from the Ministry of Health and Child Care, Zimbabwe, and this study was granted ethical approval by the University of Witwatersrand's Human Research Ethics Committee (Medical) (Clearance Certificate No. M170673).

## Results

### Descriptive Characteristics of Patients and Total Transitions Observed

From the 538 clinics, a total of 390,771 patients were seen between 1st January 2004 and 31st December 2017. Of these total patients, we excluded 197,618 (50.6%) patients with no CD4 cell counts and 129,731 (33.2%) patients with one CD4 cell count measurement. The remaining 63,422 patients of whom 65.4% were female contributed 205,711 years of total analysis time at risk and under observation from 491 health facilities form part of the analysis. The descriptive characteristics are shown in Table 1. Most patients were enrolled in district or mission hospitals (45.7%) and from facilities in the rural areas (74.7%). There was an overwhelming significant difference in the baseline characteristics by CD4 count states in this cohort, *p* < 0.05. The median follow-up time was 2.63 [interquartile range (IQR), 1.14–4.94] years, median duration between visits was 0.63 (IQR, 0.25–1.88) years, and the median number of visit was 3 (IQR, 2–4) visits. Most patients were classified in WHO clinical stage III/IV (58.6%, *n* = 36,626).

**Table 1:** Sociodemographic and clinical baseline characteristics at antiretroviral therapy (ART) initiation of all study participants from the Zimbabwe national ART program, 2004–2017.

**Variables**	**Total (%)**	**Baseline CD4 states for patients**				**Chi-square *p*-value**
		**<200**	**200 ≤ CD4 < 350**	**350 ≤ CD4 < 500**	**≥500**	
Health facility type						
Primary health care	31,150 (49.1)	4,989 (44.4)	4,801 (51.0)	9,223 (50.0)	12,137 (49.9)	< 0.0001
District/mission hospital	28,971 (45.7)	5,465 (48.6)	4,141 (44.0)	8,401 (45.6)	10,964 (45.1)	
Provincial/central hospital	3 301 (5.2)	790 (7.0)	474 (5.0)	813 (4.4)	1,224 (50.3)	
Health facility site						
Rural	47,348 (74.7)	8,401 (74.7)	7,256 (77.1)	14,109 (76.5)	17,582 (72.3)	< 0.0001
Urban	16,074 (25.3)	2,843 (25.3)	2,160 (22.9)	4,328 (23.5)	6,743 (27.7)	
Age categories (years)						
15–24	3,960 (6.2)	933 (8.3)	671 (7.1)	1,162 (6.3)	1,194 (4.9)	
25–34	17,352 (27.4)	3,347 (29.8)	2,757 (29.3)	5,158 (28.0)	6,090 (25.0)	< 0.0001
35–44	4,060 (36.1)	3,447 (36.6)	6,843 (37.1)	9,842 (40.5)	24,192 (38.1)	
45 and above	17,918 (28.3)	2,904 (25.8)	2,541 (27.0)	5,274 (28.6)	7,199 (29.6)	
Sex						
Female	41,505 (65.4)	9,068 (80.7)	6,508 (69.1)	12,027 (65.2)	13,902 (57.2)	< 0.0001
Male	21,917 (34.6)	2,176 (19.3)	2,908 (30.9)	6,410 (34.8)	10,423 (42.8)	
Educational level^*^						
None	1,499 (4.4)	266 (4.7)	250 (4.9)	445 (4.5)	538 (4.1)	
Primary	11,804 (34.9)	1,996 (35.2)	1,782 (34.9)	3,501 (35.3)	4,525 (34.4)	
Secondary	18,956 (56.0)	3,124 (55.0)	2,843 (55.6)	5,552 (56.0)	7,437 (56.5)	< 0.027
Tertiary	1,598 (4.7)	290 (5.1)	263 (4.6)	419 (4.2)	653 (5.0)	
Marital status^**^						
Single	7,604 (12.4)	1,276 (11.8)	1,144 (12.5)	2,095 (11.7)	3,089 (13.1)	
Married	37,993 (62.0)	6,580 (60.7)	5,680 (62.3)	11,460 (64.2)	14,273 (60.7)	
Widowed	11,243 (18.3)	2,250 (20.7)	1,643 (18.0)	3,083 (17.3)	4,267 (18.2)	< 0.0001
Divorced	4,494 (7.3)	741 (6.8)	655 (7.1)	1,216 (6.8)	1,882 (8.0)	
Functional status^***^						
Working	59,519 (94.5)	10,685 (95.5)	8,963 (94.7)	17,373 (94.9)	22,598 (93.6)	
Ambulatory	3,270 (5.2)	494 (4.4)	477 (5.1)	877 (4.8)	1,422 (5.9)	< 0.0001
Bedridden	193 (0.3)	11 (0.1)	21 (0.2)	43 (0.2)	118 (0.5)	
First line regimen	61,654 (97.2)	11,056 (98.3)	9,264 (98.4)	18,082 (98.1)	23,252 (95.6)	
D4T (30) + 3TC + NVP	389 (0.6)	11 (0.0)	18 (0.2)	91 (0.5)	269 (1.1)	
D4T (30) + #TC + EFV	11 (0.0)	1 (0.0)	1 (0.0)	3 (0.0)	6 (0.0)	
AZT + 3TC + NVP	486 (0.8)	91 (0.8)	59 (0.6)	150 (0.8)	186 (0.8)	
AZT + 3TC + EFT	82 (0.1)	13 (0.1)	10 (0.1)	17 (0.1)	42 (0.2)	< 0.0001
TDF + 3TC + NVP	2,919 (4.6)	303 (2.7)	255 (2.7)	907 (4.9)	1,454 (6.0)	
TDF + 3TC + EFV	57,085 (90.0)	10,358 (92.1)	8,841 (93.9)	16,807 (91.2)	21,079 (86.7)	
Other first lines	682 (1.1)	279 (2.5)	80 (0.9)	107 (0.6)	216 (0.9)	
Second line regimen	1,768 (2.8)	188 (1.7)	152 (1.6)	355 (1.9)	1,073 (4.4)	
AZT + 3TC + ATV/r	560 (0.9)	374 (1.5)	107 (0.6)	41 (0.4)	38 (0.3)	
AZT + 3TC + LPV/r	84 (0.1)	48 (0.2)	15 (0.1)	7 (0.1)	14 (0.1)	
TDF + 3TC + ATV/r	563 (0.9)	275 (1.1)	131 (0.7)	70 (0.7)	87 (0.8)	
TDF + 3TC + LPV/r	89 (0.1)	49 (0.2)	21 (0.1)	7 (0.1)	12 (0.1)	
ABC + DDI + ATV/r	73 (1)	50 (0.2)	13 (0.1)	7 (0.1)	3 (0.0)	
ABC + DDI + LPV/r	389 (0.6)	273 (1.1)	69 (0.4)	19 (0.2)	37 (0.3)	
Other second lines	683 (1.1)	220 (0.9)	106 (0.6)	81 (0.9)	276 (2.5)	
Tuberculosis status^****^						
Negative	54,474 (87.0)	9,869 (88.6)	8,172 (87.7)	15,830 (86.9)	20,603 (86.0)	
Positive	618 (1.0)	48 (0.4)	56 (0.6)	140 (0.8)	374 (1.6)	< 0.0001
Not assessed	7,524 (12.0)	1,220 (11.0)	1,090 (11.7)	2,240 (12.3)	2,974 (12.4)	
WHO clinical staging^*****^						
I/II	25,891 (41.4)	4,680 (42.0)	4,277 (46.0)	8,322 (45.9)	8,612 (36.0)	
III/IV	36,626 (58.6)	6,460 (58.0)	5,012 (54.0)	9,826 (54.1)	15,328 (63.0)	< 0.0001

* *The denominator of the proportions for this variable is33,857*.

** *The denominator of the proportions for this variable is61,334*.

*** *The denominator of the proportions for this variable is62,982*.

**** *The denominator of the proportions for this variable is62,616*.

***** *The denominator of the proportions for this variable is62,517*.

### Observed Transitions Between States

As displayed in Table 2, the 63,422 patients contributed 140,867 transitions between the follow-up period of which 12.6% (*n* = 7,995) were mortalities. The highest contribution of the observed transitions of 114,561 (81.3%) came from those patients who remained in the same state over time without making any transition to other states. At baseline, majority of the patients were in state 4 (CD4 < 200) (38.4%, *n* = 24,325) and state 3 (200 ≤ CD4 < 350) (29.1%, *n* = 18,437). Similarly, this was the picture at the end of the study; however, relative to baseline numbers, there was a non-significant decline in the total number of patients in state 3 (200 ≤ CD4 < 350) (*p* = 0.2621), while a significant decline was observed in state 4 (*p* = 0.0478). Majority of the deaths at the end of the study came from state 4 (CD4 < 200) and state 3 [200 ≤ CD4 < 350], which accounted for 27.6% (*n* = 2,208) and 56.8% (*n* = 4,541), respectively.

**Table 2:** Number of the total observed patients' transitions between the five states, the total number of patients at antiretroviral therapy (ART) initiation (“beginning state”) and the total number of patients at 31st December 2017 (and the “end state”) among ART patients in Zimbabwe national ART from 2004 to 2017.

	**To**	**State 1 (>500 cell/ml)**	**State 2 (351–500 cells/ml)**	**State 3 (200–350 cells/ml)**	**State 4 (<200 cells/ml)**	**State 5 (Death)**	**Total transitions**
**From**						
**A summary of the total observed patients' transition between the five states**						
State 1 (>500 cell/ml)		24,709	1,971	1,639	1,396	606	30,321
State 2 (351–500 cells/ml)		2,493	17,169	1,691	1,134	640	23,127
State 3 (200–350 cells/ml		3,459	2,606	28,645	1,763	2,208	38,681
State 4 (<200 cells/ml)		2,879	2,343	2,932	36,043	4,541	48,738
Total		33,540	24,089	34,907	40,336	7,995	140,867
**Total number of patients by CD4 state at ART initiation and the end of follow-up (denominator** **=** **63,422)**						
Beginning state *n* (%)		11,244 (17.7)	9,416 (14.9)	18,437(29.1)	24,325 (38.4)	–	63,422
End state *n* (%)		14,463 (22.8)	10,378 (16.4)	14,663 (23.1)	15,923 (25.1)	7,995 (12.61)	55,427 (alive) 7,995 (dead)

Immune recovery is observed when a patient makes a transition from lower CD4 cell counts states to higher CD4 cell counts states (particularly 350 ≤ CD4 < 500 state to CD4 ≥ 500 state, 200 ≤ CD4 < 350 state to 350 ≤ CD4 < 500 state and CD4 < 200 state 4 to 200 ≤ CD4 < 350 state), while immune deterioration is experienced if a patient makes a transition from higher CD4 cell count states to lower CD4 cell count states (particularly CD4 ≥ 500 state to 350 ≤ CD4 < 500 state, 350 ≤ CD4 < 500 state to 200 ≤ CD4 < 350 state, and 200 ≤ CD4 < 350 state to CD4 < 200 state). There were more transitions (*n* = 8,031) from lower CD4 cell counts states to higher CD4 cell counts states (state 2 to 1 = 2,493, state 3 to 2 = 2,606, and state 4 to 3 = 2,932) as compared to higher CD4 cell counts states to lower CD4 cell counts states transitions of the corresponding reversible transitions (*n* = 5,425). This result is an indication of immune recovery in this cohort.

### Time Homogenous Transition Rates and Probabilities

The transition rates and probabilities were estimated using the time-homogenous multistate Markov model incorporating the semiparametric Cox survival function, and results are displayed in Table 3. Generally, there were higher transition rates from lower CD4 cell count states to lower CD4 cell counts states compared to the reversible corresponding transitions. Results show that moving from state 2 (350 ≤ CD4 < 500) to state 1 (CD4 ≥ 500) was 1.49 (0.16085/0.10783) times more likely than moving from state 1 (CD4 ≥ 500) to 2 (350 ≤ CD4 < 500); hence, a high probability of immune recovery. Patients in state 2 (350 ≤ CD4 < 500) were 1.38 (0.11264/0.08188) times more likely to move to state 3 (200 ≤ CD4 < 350) compared to moving from state 3 (200 ≤ CD4 < 350) to state 2 (350 ≤ CD4 < 500). This finding was a clear indication of immune deterioration between the two states. Transition rate from state 4 (CD4 < 200) to state 3 (200 ≤ CD4 < 350) was 1.02 (0.05261/0.05147) times more likely compared to the transition from state 3 (200 ≤ CD4 < 350) to state 4 (CD4 < 200) indicating immune recovery from state 4 (CD4 < 200), but this was not statistically significant.

**Table 3:** Estimates of transition rates (intensities) per person-years and probability matrices and 95% confidence intervals (CI) for the time-homogenous multistate Markov model among antiretroviral therapy (ART) patients in Zimbabwe national ART from 2004 to 2017.

**Transitions**	**Crude transition intensity (95% CI)**	**Covariates adjusted transition intensity(95% CI)**	**Transition probabilities(95% CI)**	**The probability that each state is next**
1 → 1	−0.26548 (−0.273 to −0.258)	−0.26950 (−0.278 to −0.261)	0.779 (0.772 to 0.784)	0
1 → 2	0.10928 (0.104 to 0.115)	0.10783 (0.102 to 0.114)	0.083 (0.08 to 0.086)	0.41162 (0.397 to 0.427)
1 → 3	0.07823 (0.074 to 0.083	0.08184 (0.0772 to 0.087)	0.067 (0.063 to 0.070)	0.29468 (0.280 to 0.309)
1 → 4	0.05980 (0.056 to 0.063)	0.06313 (0.059 to 0.067)	0.052 (0.050 to 0.055)	0.22526 (0.214 to 0.238)
1 → 5	0.01817 (0.016 to 0.021)	0.01669 (0.014 to 0.019)	0.020 (0.018 to 0.022)	0.06844 (0.061 to 0.077)
2 → 1	0.18073 (0.173 to 0.189)	0.16085 (0.153 to 0.169)	0.136 (0.132 to 0.142)	0.45731 (0.443 to 0.472)
2 → 2	−0.39520 (−0.406 to −0.384)	−0.37098 (−0.382 to−0.360)	0.686 (0.679 to 0.692)	0
2 → 3	0.11767 (0.112 to 0.124)	0.11264 (0.106 to 0.119)	0.092 (0.088 to 0.096)	0.29775 (0.285 to 0.312)
2 → 4	0.06960 (0.065 to 0.074)	0.07160 (0.067 to 0.077)	0.058 (0.055 to 0.061)	0.17613 (0.165 to 0.186)
2 → 5	0.02719 (0.024 to 0.031)	0.02589 (0.023 to 0.029)	0.027 (0.025 to 0.030)	0.06881 (0.061 to 0.077)
3 → 1	0.09501 (0.091 to 0.099)	0.08428 (0.080 to 0.088)	0.080 (0.077 to 0.083)	0.35266 (0.340 to 0.365)
3 → 2	0.08723 (0.083 to 0.091)	0.08188 (0.078 to 0.086)	0.068 (0.065 to 0.071)	0.32376 (0.312 to 0.336)
3 → 3	−0.26942 (−0.276 to −0.263)	−0.24886 (−0.256 to −0.243)	0.772 (0.768 to 0.776)	0
3 → 4	0.05397 (0.051 to 0.057)	0.05147 (0.049 to 0.055)	0.047 (0.045 to 0.045)	0.20032 (0.190 to 0.210)
3 → 5	0.03321 (0.031 to 0.035)	0.03122 (0.029 to 0.034)	0.032 (0.031 to 0.034)	0.12325 (0.116 to 0.131)
4 → 1	0.04814 (0.046 to 0.051)	0.04363 (0.041 to 0.046)	0.044 (0.042 to 0.045)	0.22794 (0.218 to 0.238)
4 → 2	0.04584 (0.044 to 0.048)	0.04360 (0.041 to 0.046)	0.038 (0.036 to 0.040)	0.21708 (0.207 to 0.228)
4 → 3	0.05793 (0.055 to 0.061)	0.05261 (0.050 to 0.055)	0.049 (0.048 to 0.051)	0.27430 (0.263 to 0.285)
4 → 4	−0.21118 (−0.215 to −0.207)	−0.19535 (−0.200 to −0.191)	0.814 (0.810 to 0.817)	0
4 → 5	0.05928 (0.057 to 0.061)	0.05551 (0.053 to 0.058)	0.055 (0.053 to 0.057)	0.28069 (0.272 to 0.290)

We estimated the probabilities for which state is next after the currently occupied state. The results show that an individual in state 1 (CD4 ≥ 500) had a probability of 41.2% to move to state 2 (350 ≤ CD4 < 500); an individual in state 2 (350 ≤ CD4 < 500) had a 45.7% probability to move to state 1 (CD4 ≥ 500); an individual in state 3 (200 ≤ CD4 < 350) had 35.3% probability to move to state 1 (CD4 ≥ 500); and an individual in state 4 (CD4 < 200) had 28.1% probability of death. The cumulative probability of moving from higher CD4 cell counts states to lower CD4 cell counts states increased over time. The probability of moving from state 1 (CD4 ≥ 500) to state 2 (350 ≤ CD4 < 500) changed from 8.3% at 1 year to 16.2% at 6 years; state 2 (350 ≤ CD4 < 500) to state 3 (200 ≤ CD4 < 350) transition changed from 9.2% at 1 year to 20.2% at 6 years and state 3 (200 ≤ CD4 < 350) to state 4 (CD4 < 200) transition changed from 4.7% at 1 year to 15.3% at 6 years. Similarly, the probabilities of moving from lower CD4 cell counts states to higher CD4 cell counts states increased over time. The probability of moving from state 2 (350 ≤ CD4 < 500) to state 1 (CD4 ≥ 500) changed from 13.6% at 1 year to 26.2% at 6 years; state 3 (200 ≤ CD4 < 350) to state 2 (350 ≤ CD4 < 500) transition changed from 6.8% at 1 year to 14.7% at 6 years and state 4 (CD4 < 200) to state 3 (200 ≤ CD4 < 350) transition changed from 4.5% at 1 year to 14.5% at 6 years.

### Time Inhomogenous Mortality Rates

**Figure 2:**
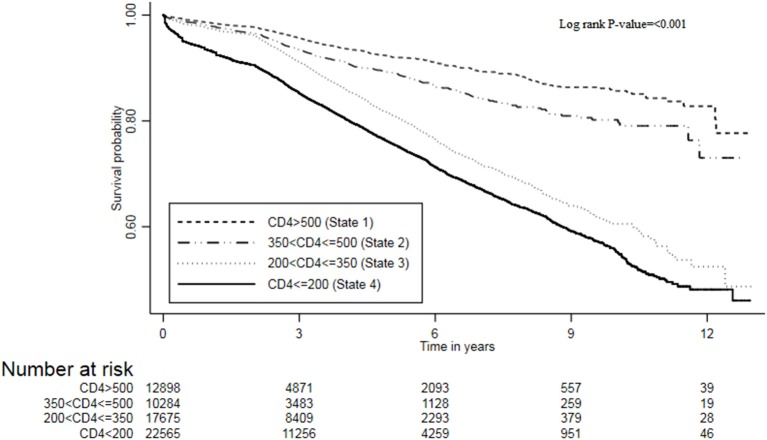
The survival plot of ART patients from the Zimbabwe national ART program stratified by CD4 cell counts states, 2004–2017.

The transition rates for mortality were also estimated, and results are shown in Table 4. The overall mortality rate in this cohort was 3.9 (95% CI, 3.8–4.0) per 100 person-year. Stratifying by the CD4 states, the mortality rates per 100 person-years increased with a decrease in CD4 cell counts: state 1 (CD4 ≥ 500) (rate = 1.8; 95% CI, 1.1–2.1), state 2 (350 ≤ CD4 < 500) (rate = 2.7; 95% CI, 2.4–3.1), state 3 (200 ≤ CD4 < 350) (rate = 3.3; 95% CI, 3.1–3.8), and state 4 (CD4 < 200) (rate = 5.9; 95% CI, 5.7–6.1). Hence, the mortality burden was highest in state 4 (CD4 < 200) compared to other states, and these mortality rates were significantly different (log rank test *p* < 0.001). The Kaplan–Meier curve further confirmed the survival probabilities of this cohort stratified by state, that mortality risk increases with a decrease in CD4 cell count (Figure 2). However, the fundamental difference was between the mortality in state 3 (200 ≤ CD4 < 350) and state 4 (CD4 < 200) vs. the mortality in state 1 (CD4 ≥ 500), and state 2 (350 ≤ CD4 < 500).

**Table 4:** Estimated time-varying mortality rates per person-years and 95% confidence intervals for the time-inhomogenous multistate Markov model among ART patients in Zimbabwe national ART from 2004 to 2017.

**Time period**	**Time-varying transition rates (95% confidence interval)**					
	**Overall mortality rate**	**State 1 to 5**	**State 2 to 5**	**State 3 to 5**	**State 4 to 5**	**Log rank *p-*value**
0 < time ≤ 1	0.3512 (0.3368–0.3661)	0.1006 (0.0863–0.1172)	0.1490 (0.1292–0.1719)	0.2790 (0.2534–0.3071)	0.7609 (0.7225–0.8013)	<0.0001
1 < time ≤ 2	0.0505 (0.0472–0.0540)	0.0186 (0.0147–0.0234)	0.0291 (0.0239–0.0355) |	0.0383 (0.0330–0.0444)	0.1010 (0.0925–0.1102)	<0.0001
2 < time ≤ 3	0.0591 (0.0563–0.0621)	0.0245 (0.0206–0.0290)	0.0287 (0.0244–0.0338)	0.0687 (0.0632–0.0747)	0.0885 (0.0825–0.0950)	<0.0001
3 < time ≤ 4	0.0416 (0.0392–0.0441)	0.02000.0162–0.0247)	0.0186 (0.0148–0.0233)	0.0441 (0.0400–0.0486)	0.0581 (0.0535–0.0631)	<0.0001
4 < time ≤ 5	0.0312 (0.0291–0.0334)	0.0135 (0.010–0.0179)	0.0162 (0.0121–0.0217)	0.0312 (0.0277–0.0351)	0.0412 (0.0375–0.0453)	<0.0001
5 < time ≤ 6	0.0221 (0.0204–0.0240)	0.0076 (0.0053–0.0109)	0.0133 (0.0099–0.0180)	0.0214 (0.0185–0.0247)	0.0310 (0.0278–0.0346)	<0.0001
6 < time ≤ 7	0.0160 (0.0145–0.0178)	0.0079 (0.0057–0.0111)	0.0092 (0.0061–0.0137)	0.0160 (0.0133–0.0193)	0.0215 (0.0188–0.0247)	<0.0001
7 < time ≤ 8	0.0112 (0.0097–0.0128)	0.0041 (0.0025–0.0066)	0.0052 (0.0029–0.0095)	0.0117 (0.0088–0.0155)	0.0159 (0.0134–0.0189)	<0.0001
8 < time ≤ 9	0.0108 (0.0092–0.0128)	0.0050 (0.0030–0.0083)	0.0044 (0.0021–0.0093)	0.0132 (0.0094–0.0186)	0.0142 (0.0116–0.0175)	<0.0001
9 < time ≤ 10	0.0070 (0.0055–0.0089)	0.0014 (0.0005–0.0044)	0.0019 (0.0048–0.0076)	0.0082 (0.0049–0.0136)	0.0103 (0.0077–0.0138)	0.0001
10 < time ≤ 11	0.0066 (0.0050–0.0088)	0.0021 (0.0008–0.0056)	0.0021 (0.0005–0.0084)	0.0072 (0.0036–0.0145)	0.0104 (0.0074–0.0147)	0.0005
11 < time ≤ 12	0.0034 (0.0020–0.0058)	0.0015 (0.0004–0.0061)	0.0046 (0.0012–0.0184)	0.0066 (0.0025–0.0176)	0.0033 (0.0014–0.0080)	0.2154
12 < time ≤ 13	0.0030 (0.0011–0.0081)	0.0058 (0.0014–0.0230)	0	0.0031 (0.0004–0.0220)	0.0021 (0.0003–0.0151)	0.7305

In general, the time-varying mortality rates decrease with an increase in time since ART. The cohort experienced high mortality rates in the first year of ART initiation averaging at 3.5 (95% CI, 3.4–3.7) per 100 person-years. There was a sharp drop (seven-fold) in mortality rate from first to the second year [hazard ratio (HR) = 6.95(0.3512/0.0505); 95% CI, 6.78–7.14]. Gradually, the mortality rates further decrease over time by the end of the follow-up period. Mortality patterns across states followed this similar trend to the overall pattern. In the first 3 years, mortality rates had an inverse relationship with the CD4 cell counts, and there was an overwhelming difference in these rates between the states.

This study forecasted the total length spent in each of the CD4 states by HIV patients on ART before death and estimated the mean sojourn (holding) time for each state as shown in Table 5. The results show that, when an individual enters state 4 (CD4 < 200), the time he or she spends in this state for a single stay before moving to another state was estimated to be 4.74 (4.64–4.83) years on average. This result could be linked to the time taken by a patient in this state to respond to ART and subsequently boost immunity since this is the worst state in our HIV progression model. Since the holding times for all states are relatively long, therefore, HIV disease progression in this cohort was relatively slow.

**Table 5:** Estimates of mean sojourn time and the total length of stay for the time-homogenous multistate Markov model among antiretroviral therapy (ART) patients in Zimbabwe national ART from 2004 to 2017.

**States**	**Mean sojourn time**		**The total length of stay (years)**
	**Estimate (95% CI) (years)**	**Standard error**	
State 1 (>500 cell/μl)	3.77 (3.66–3.88)	0.054	11.33
State 2 (351–500 cells/μl)	2.53 (2.46–2.60)	0.036	5.51
State 3 (200–350 cells/μl)	3.71 (3.63–3.80)	0.042	7.17
State 4 (< 200 cells/μl)	4.74 (4.64–4.82)	0.047	6.86

It was also of interest to forecast the total length of stay for states 1–4 before death, which is and quite informative in the presence of reversible transitions. The results show that an individual will stay 11.3 years in state 1 (CD4 ≥ 500), 5.5 years in state 2 (350 ≤ CD4 < 500), 7.2 years in state 3 (200 ≤ CD4 < 350) and 6.9 years in state 4 (CD4 < 200) before death. In general, these results reflected that an HIV patient on ART is expected to spend more time in the highest CD4 cell counts state compared to other states.

### Covariates Effects on Immune Recovery and Deterioration Transition Rates

We further included time-independent covariates (health facility level, ART initiation period, and sex) and age in the multistate Cox proportional hazard model, and the results are displayed in Table 6. This model was a better fit using a likelihood ratio test compared to the model without covariates (*p* < 0.001). Adjusting for other covariates, the higher levels of health facility are more likely to have patients moved from lower to higher CD4 cell count states. Provincial or central hospital individuals were predominantly more likely to move from state 4 (CD4 < 200) to state 3 (200 ≤ CD4 < 350) (HR = 8.30; 95% CI, 6.64–10.36) followed by the state 3 (200 ≤ CD4 < 350) to state 2 (350 ≤ CD4 < 500) transition (HR = 8.04; 95% CI, 6.41–10.10) compared to PHC patients. This means that patients at the provincial or central hospital had a high probability of immune deterioration once they are on ART compared to PHC patients. For district or mission hospital patients, state 3 (200 ≤ CD4 < 350) to state 2 (350 ≤ CD4 < 500) was the predominant transition (HR = 4.41; 95% CI, 3.96–4.87), followed by the state 4 (CD4 < 200) to state 3 (200 ≤ CD4 < 350) transition (HR = 3.97; 95% CI, 3.61–4.37), compared to PHC patients. Similarly, this was a positive indication of immune recovery for patients in district or mission hospital compared to PHC patients.

**Table 6:** Multiple variable estimates of the hazard ratios and 95% confidence intervals from the time-homogenous multistate Cox proportional hazard model among antiretroviral therapy (ART) patients in Zimbabwe national ART from 2004 to 2017.

**Transitions**	Health facility type^*^		Age categories in years^**^			Sex ^***^	ART initiation period^****^	
	**District/mission**	**Provincial/central**	**15–24**	**35–44**	**45+**	**Male**	**2008–2012**	**2013–2017**
1 → 2	**2.36 (2.12–2.62)**	**2.10 (1.69–2.62)**	**0.78 (0.61–0.99)**	**1.25 (1.10–1.41)**	**1.35 (1.18–1.54)**	**1.15 (1.01–1.28)**	**0.78 (0.67–0.93)**	0.90 (0.76–1.07)
1 → 3	**1.28 (1.14–1.43)**	**0.58 (0.39–0.85)**	0.80 (0.62–1.04)	1.11 (0.96–1.27)	**1.32 (1.14–1.53)**	1.04 (0.91–1.19)	**1.65 (1.31–2.10)**	**1.62 (1.27–2.07)**
1 → 4	1.01 (0.90–1.14)	**0.33 (0.22–0.50)**	0.90 (0.68–1.21)	1.05 (0.91–1.22)	**1.32 (1.13–1.54)**	0.91 (0.78–1.05)	**0.35 (0.30–0.42)**	**0.25 (0.21–0.31)**
1 → 5	**1.48 (1.19–1.84)**	**1.89 (1.32–2.67)**	**3.71 (2.90–4.76)**	0.68 (0.51–0.91)	0.94 (0.70–1.24)	**1.56 (1.26–1.92)**	**3.26 (2.07–5.14)**	**3.02 (1.87–4.89)**
2 → 1	**2.84 (2.57–3.13)**	**3.12 (2.54–4.36)**	0.98 (0.80–1.19)	1.05 (0.94–1.18)	1.06 (0.93–1.20)	**0.57 (0.51–0.64)**	**0.56 (0.47–0.67)**	**0.54 (0.45–0.64)**
2 → 3	**3.18 (2.83–3.58)**	**3.33 (2.54–4.36)**	0.74 (0.54–1.00)	**1.31 (1.14–1.51)**	**1.56 (1.34–1.81)**	**1.23 (1.10–1.38)**	0.88 (0.71–1.08)	**0.39 (.30–0.52)**
2 → 4	**1.27 (1.11–1.46)**	1.20 (0.83–1.73)	0.76 (0.52–1.10)	**1.25 (1.05–1.48)**	**1.33 (1.11–1.60)**	1.10 (0.96–1.27)	**0.64 (0.49–0.83)**	**0.54 (0.43–0.67)**
2 → 5	**1.51 (1.17–1.95)**	**3.36 (2.05–5.52)**	**1.66 (1.09–2.53)**	0.87 (0.63–1.20)	**1.37 (1.01–1.86)**	0.89 (0.69–1.15)	**2.67 (1.20–5.95)**	**4.89 (2.22–10.79)**
3 → 1	**2.54 (2.32–2.79)**	**4.28 (3.45–5.30)**	1.12 (0.94–1.36)	0.91 (0.81–1.01)	0.92 (0.81–1.03)	**0.46 (0.41–0.52)**	1.11 (0.87–1.43)	1.11 (0.86–1.44)
3 → 2	**4.41 (3.96–4.87)**	**8.04 (6.41–10.10)**	0.79 (0.61–1.03)	**1.24 (1.10–1.40)**	**1.35 (1.18–1.54)**	0.96 (0.87–1.06)	**0.70 (0.57–0.87)**	**0.66 (0.53–0.82)**
3 → 4	**2.62 (2.34–2.93)**	**2.28 (1.59–3.28)**	0.98 (0.73–1.32)	**1.29 (1.12–1.50)**	**1.53 (1.32–1.79)**	**1.67 (1.49–1.86)**	**0.43 (0.35–0.54)**	**0.51 (0.40–0.63)**
3 → 5	1.16 (0.99–1.13)	1.25 (0.73–2.16)	**1.71 (1.32–2.21)**	0.93 (0.77–1.08)	1.18 (0.99–1.42)	**1.32 (1.15–1.51)**	1.69 (0.99–2.85)	**4.14 (2.47–6.96)**
4 → 1	**2.29 (2.05–2.55)**	0.63 (0.34–1.17)	**1.39 (1.09–1.77)**	1.01 (0.89–1.16)	**1.20 (1.04–1.38)**	**0.36 (0.31–0.41)**	**0.28 (0.24–0.34)**	**0.18 (0.15–0.22)**
4 → 2	**2.70 (2.41–3.03)**	**4.88 (3.55–6.71)**	0.82 (0.60–1.11)	1.07 (0.94–1.24)	**1.20 (1.04–1.40)**	**0.72 (0.64–0.81)**	1.01 (0.77–1.33)	0.89 (0.67–1.18)
4 → 3	**3.97 (3.61–4.37)**	**8.30 (6.64–10.36)**	0.84 (0.64–1.09)	1.11 (0.99–1.25)	**1.25 (1.10–1.42)**	**1.28 (1.17–1.41)**	0.95 (0.76–1.19)	**1.42 (1.13–1.78)**
4 → 5	**1.60 (1.49–1.73)**	**2.23 (1.80–2.74)**	**1.71 (1.47–1.98)**	0.95 (0.86–1.04)	1.08 (0.98–1.20)	**1.32 (1.23–1.42)**	**2.18 (1.69–2.82)**	**9.15 (7.12–11.79)**

*The covariates included in the adjusted model were those with complete observation information and considered possible risk factors for the individual transitions*.

* *Reference: Primary health care facilities*.

** *Reference: 25–34 years age group*.

*** *Reference: female patients*.

**** *Reference: 2004–2007 time period*.

*Bold face values are significant at 5%*.

Adjusting for other covariates, age was generally associated with immune deterioration transitions (CD4 ≥ 500 state to 350 ≤ CD4 < 500 state, 350 ≤ CD4 < 500 state to 200 ≤ CD4 < 350 state, and 200 ≤ CD4 < 350 state to CD4 < 200 state). Compared to the 25–34 years age group, there was no significant difference in immune deterioration transitions. However, the results showed that the older the patient, the more likely he or she is to become immune deteriorated. This result was observed in elderly patients (45+ years) with a pronounced risk of immune deterioration across age groups. With reference to 25–34 years age group, both the 35–44 years and the 45+ years age groups were predominantly more likely to move from state 2 (350 ≤ CD4 < 500) to state 3 (200 ≤ CD4 < 350) transition (HR = 1.31; 95% CI, 1.14–1.51) and (HR = 1.56; 95% CI, 1.34–1.81), respectively. Holding other covariates constant, sex was significantly associated with immune deterioration transitions. Male patients had an increased risk of immune deterioration compared to female patients: state 1 (CD4 ≥ 500) to state 2 (350 ≤ CD4 < 500) (HR = 1.15; 95% CI, 1.01–1.28), state 2 (350 ≤ CD4 < 500) to state 3 (200 ≤ CD4 < 350) (HR = 1.23; 95% CI, 1.10–1.38) and state 3 (200 ≤ CD4 < 350) to state 4 (CD4 < 200) (HR = 1.67; 95% CI, 1.49–1.86). Moving from state 3 (200 ≤ CD4 < 350) to state 4 (CD4 < 200) was predominant in male compared to female patients.

### Covariates Effects on Mortality Rates

In overall, mortality was high among patients in state 4 (CD4 < 200) in this cohort. The mortality risk was pronounced among patients in provincial or central hospitals than those in district hospitals if in state 1 (CD4 ≥ 500) (HR = 1.89; 95% CI, 1.32–2.67), state 2 (350 ≤ CD4 < 500) (HR = 3.36; 95% CI, 2.05–5.52), state 3 (200 ≤ CD4 < 350) (HR = 1.25; 95% CI, 0.73–2.16), and state 4 (CD4 < 200) (HR = 2.23; 95% CI, 1.80–2.74). State 2 (350 ≤ CD4 < 500) mortality risk was predominant in the provincial or central hospitals. This means that PHC facilities had a low risk of mortality in this cohort compared to both a higher level of care facilities. Interestingly, the mortality risk was much more pronounced among the 15–25 years age groups than other age groups. The mortality risk for state 1 (CD4 ≥ 500) was 3.71 (95% CI, 2.90–4.76), state 2 (350 ≤ CD4 < 500) (HR = 1.66; 95% CI, 1.09–2.53), state 3 (200 ≤ CD4 < 350) (HR = 1.71; 95% CI, 1.32–2.21, and state 4 (CD4 < 200) (HR = 1.71; 95% CI, 1.47–1.98). Patients who were aged 45 years and above were more likely to immune deteriorate compared to 25–34 years age group: HR, 1.35; 95% CI, 1.18–1.54; HR, 1.56; 95% CI, 1.34–1.81, and HR, 1.53; 95% CI, 1.32–1.79 for state 1 to 2, state 2 to 3, and state 3 to 4, respectively. Male patients were more likely to die compared to female patients: state 1–5 (HR = 1.56; 95% CI, 1.26–1.92), state 3–5 (HR = 1.32; 95% CI, 1.15–1.51), and state 4 to 5 (HR = 1.32; 95% CI, 1.23–1.42). Considering the ART initiation period, mortality risks were pronounced among patients who initiated ART in 2013–2017: state 2 (350 ≤ CD4 < 500) (HR = 4.89; 95% CI, 2.22–10.79), state 3 (200 ≤ CD4 < 350) (HR = 4.14; 95% CI, 2.47–6.96), and state 4 (CD4 < 200) (HR = 9.15: 95% CI, 7.12–11.79).

## Discussion

This study's objective was to describe HIV disease progression (immune deterioration) and immune recovery among adult patients on ART in Zimbabwe using patient-level data after ART decentralization. This study made use of semiparametric time homogenous and time inhomogenous multistate Markov models based on four CD4 cell counts intermediate transient states and mortality as the absorbing state. This study was a quantitative secondary data analysis of the routinely collected patient-level data through ePMS among HIV-infected patients on ART in Zimbabwe between 2004 and 2017. The study findings were comparable to other earlier studies and indicated a poor immune recovery in PHC facilities compared to higher levels of care facilities. This study observed significant findings to evaluate HIV disease progression and immune recovery based on CD4 cell counts among ART patients between 2004 and 2017 in Zimbabwe after the decentralization of ART services. The estimated mortality rate of 3.9 per 100 person-years is low and patients in state 4 (CD4 < 200) had the highest risk of death (5.9 per 100 person-years on average) compared to other states. This finding was evident throughout in the time-varying analysis of rates. The high rates in state 4 (CD4 < 200) were consistent over time; however, there was a sharp drop by seven-fold from 1 to 2 years since ART initiation. There finding of high rates in lower CD4 cell count states is comparable to finding from previous work in India and South Africa (13, 14). Immune deterioration pronounced in patients aged 45 years and above, provincial or central hospital levels of care and male patients. However, immune recovery was also observed in this cohort since there were higher transitions and transition rates from lower CD4 cell counts states to higher CD4 cell counts states. Moreover, patients in the high levels of care (district and provincial or central hospitals) had an increased probability of immune recovery compared to PHC facilities; however, mortality was high in the high levels of care. Male patients had an increased risk of mortality compared to female patients in this cohort.

Generally, there was a gradual improvement in CD4 cell count after ART initiation. This result was evident by the higher immune recovery rates compared to immune deterioration rates. This is an indication of effective ART treatment to HIV infected individuals and that if ART is initiated at early phases of HIV infection (with baseline CD4 cell count at least 350), immune recovery and reduced progression can be achieved since the immune system is intact. This matches the findings reported in South Africa in a similar population (17). This study also found out that a patient in state 1 (CD4 ≥ 500) is estimated to spend 11.3 years in higher CD4 cell count state before death, which is similar to other findings (11). This means that if individuals have a good immunity which can be attributed to the ART regimen efficacy, they tend to live longer than those with weak immunity. This study further found that the probability of mortality increases with a decrease in CD4 cell count, which concurs with findings from similar settings (17, 30). This is explained by the fact that being in an AIDS-defining stage leads to the highest probability of mortality. The highest mean sojourn time was in state 4 (CD4 < 200) compared to other states. This finding can be explained by the fact that patients with deteriorated immunity (low CD4 cell count) take a longer time to respond to treatment and boost their immunity before moving to lower states (31). Research has shown that CD4 cell count may remain unchanged despite the suppressed viral load due to weak CD4cell recovery in other patients (32). This is the limitation of using CD4 cell count; hence, use of viral load in monitoring the efficacy of ART treatment is recommended (33).

We found that the higher the level of care, the better the probability of immune recovery. Patients enrolled in either provincial or central hospitals and district facilities had an increased probability of immune recovery relative to those in PHC. The risk of immune recovery increased with an increase in care regardless of the immune status of the patient. This result can be supported by high resources through government channels or donor-funded and skilled personnel at the high levels of care (21). As much as patients prefer PHC facility for ART services because of reduced transport cost, easy to access (20), they are most likely understaffed. In addition, PHC are at times overburdened resulting in a high patient care turnaround time (34–37). Surprisingly, we observed relatively high mortality rates among patients enrolled in higher levels of care since one would anticipate the opposite to occur. However, this finding could be explained by either the referral system of patients within the patient care cascade or “silent-transfer” of patients from one health facility to another seeking better care (38–40). This means that the tertiary health facilities were more likely to receive patients who are more seriously ill and with a greater likelihood of death (38, 41, 42).

As we accounted for interindividual variability effects to get more insight on HIV disease progression in this cohort, we found that HIV patients who were aged 15–24 years at ART initiation tend to have a higher mortality than patients aged 25–34 years, and the progression to death was much more pronounced if a patient was coming from state 1 (CD4 ≥ 500) or state 2 (350 ≤ CD4 < 500). This finding supports other earlier studies which showed that adolescents are heavily burdened by chronic complications; hence, require high level of patients management (43). In addition, this group is prone to stigma, vulnerable, and prone to various chronic comorbidities as well as being and the transitional stage of becoming independent without much parental care. Intensifying community-based support for caregivers can help reduce poor health outcomes in adolescence (44). However, more research is required to further confirm this observed association in our study. Patients aged 45 years and above showed a higher risk of immune deterioration compared to younger patients (25–34 years), which was similar to other studies which reported that younger people have a higher probability of immune recovery than the elderly (11, 12). In addition, this could be explained by the immune response in older patients is weak compared to young people, that is, the capacity to generate CD4 cell counts and suppress viral load is reduced in elderly patients (45). Moreover, this could be explained by the fact that this age group is highly associated with of non-communicable diseases like hypertension and diabetes. Managing an HIV patient with multiple comorbidities is known to be complex and also intake of different drugs results in overlapping drug toxicity and lowering of the ART drug effect (35). As a result, most patients with comorbidities (communicable or non-communicable diseases) may either default ART treatment or ART drug becomes less effective due to the presents of other medications an individual is on; therefore, these patients subsequently get worse. These results confirm the need for test and treat regardless of disease stage and age which have much positive influence in patients aged 45 years and above (46, 47).

In our study, we found that male patients had higher rates of immune deterioration. This was quite pronounced on the transition from state 3 (200 ≤ CD4 < 350) to state 4 (CD4 < 200). In addition to this, we also observed poor survival outcomes among male patients. This finding is consistent with other results from Shoko and Chikobvu (17) who found out that men were six times more likely to move to higher CD4 cell count state. Another study which supports this result reported that male patients gain fewer CD4 cell counts as compared to female patients, and they have an increased immunological non-response than female patients (48). However, this finding contradicts other earlier studies which documented that gender difference does not exhibit any significant differences in HIV disease progression (11, 12). The participants in this study were predominantly female, and this could mirror the fact that female patients have better involvement in HIV issues and their health-seeking behavior compared to male patients. Female patients have multiple entry points in HIV care like efficient linkage of ART treatment in antenatal clinics and prevention-of-mother-to-child programs which results are better immune recovery than male patients (48). Male involvement in HIV care strategies needs to be enhanced to compliment female role in HIV prevention (49–53). Therefore, there is a need to scale up HIV testing rate among men and intensify repeated testing and increasing acceptance of HIV care linkages. With the critical societal role played by men, they improve decision making within a household and society at large if they are fully involved in HIV programs (54). There is need to intensify existing strategies like male circumcision, self-testing, HIV programs at workplaces, and recreational places and also come up with flexible clinic hours and conditions which accommodate men like shortening clinic turnaround time and increase privacy (48).

Our results should be viewed in light of some limitations. The dataset used had incomplete information especially in the clinical parameters which resulted in dropping off a considerable portion of the data. In addition, this study could not adjust for ART adherence, which is an important issue in HIV disease progression since it directly associated with the probability of moving to a lower CD4 cell count state if a patient fails to adhere to treatment. This study also considered patients from ART centers linked to the ePMS; this might have caused overestimation or underestimation of the transition intensities reported in this study. The analysis was solely based on the time homogenous assumption which is much more useful in the presence of heavy right censoring. Earlier studies have shown that, if a patient on ART is virally suppressed, if there is no treatment uptake violation, that patient is likely to continue recovering well. However, this violates the Markov and memory loss properties of these models, and this limitation affects the time-homogenous Markov process models. Other assumptions like non-Markovian, semi-Markovian, or hidden Markovian can be explored incorporating interval censoring and assuming time-varying effects. This model could not account for frailty terms to explain unobserved individual heterogeneity and spatial effects to show regions with an increased likelihood for a particular transition.

Moreover, this study covers the period in which ART initiation guidelines were changed three times; hence, there could be some bias in the estimates. In addition, the period covered is mainly when the country was conducting targeted differential monitoring, whereby most of the patients who had their CD4 measurement taken were mostly those carried out on the discretion of the physician. Authors acknowledge the measurement error (55) associated with CD4 cell counts in ART monitoring since a patient's measurement may indicate a lower CD4 when in fact the patients had recovered, hence the switch to use viral load in ART monitoring.

There could be possible participant inclusion bias in this study since we excluded those who were lost to follow-up (LTFU) ending up with a subsample. The exclusion of this group was to have a less complicated model with fewer states since this group would be a stand-alone compartment. However, this may have impacted in the generalizability of our research findings in that the model used is not a complete picture of the transition patterns in an ART program as some of the exit points have been excluded. Majority of the patients who became LTFU were mainly those who were very sick (with a CD4 < 200) and if tracked there could be a possibility that some of them would have died (56). The implications of such a LTFU pattern normally lead to data missing not at random in longitudinal time to event studies. Had we included the LTFU group and right censored them in their last observed states, this would have caused an upward bias of the Kaplan–Meier curve, which at times may affect the generalizability of the findings (57). In future studies, it would be essential to include the LTFU and withdrawals states in the model to have detailed transition patterns of these outcomes in an ART program. Our data could not allow us to estimate transitions to AIDS since the information was not available and exhaustively adjust for comorbidities which might be linked to the observed transition patterns in this cohort other than tuberculosis. However, tuberculosis was not included as a covariate because of the highly computational intensive of this reasonably huge dataset if many covariates are added. Hence, we restricted our analysis to demographic covariate so that we attain convergence. A notable limitation in this study is the low mortality rate of which most deaths were for those patients who initiated ART in the 2013–2017-year period. The plausible explanation for this could be an issue of a biased dataset in terms of capturing patient's information. It is most likely that the majority of the deaths that occurred earlier may have been lost during data capturing from patients files to the electronic database since this was a retrospective exercise. Thus, we are most likely to have the long-term survivors from the early period.

## Conclusion

Multistate models are crucial in providing the general disease trajectories through intermediates states to alert program response before an adverse event occurs. Our findings have significant implication in the continuum of HIV care. It is prudent to target early ART treatment initiation to prevent subsequent immune deterioration. Once this is achieved, survival outcomes and quality of life can be improved with the subsequent reduction in opportunistic infections. Strengthening of PHC facilities in ART is imperative in decentralization environment. More aggressive male involvement strategies should be enhanced to strengthen male involvement in HIV care, and adolescents/young adult management has to be upscaled to prevent ART defaulting and avert poor health outcomes.

## Data Availability Statement

The dataset used for this study can be found through an application process from the Ministry of Health and Child Care in Zimbabwe which is the custodian of the ePMS data through the AIDS/TB Unit who manages and oversees the ePMS data collection process.

## Author Contributions

ZM cleaned and analyzed the data and drafted the manuscript. JT and TC reviewed the manuscript and advised on analysis. EM guided and oversaw the analysis and reviewed the manuscript. All authors reviewed the final manuscript before submission.

### Conflict of Interest

The authors declare that the research was conducted in the absence of any commercial or financial relationships that could be construed as a potential conflict of interest.
