# Supplementary material for: HIV Disease Progression Among Antiretroviral Therapy Patients in Zimbabwe: A Multistate Markov Model
Source: Front Public Health. 2019 Nov 15;7:326. doi: 10.3389/fpubh.2019.00326 (PMC6873884; doi:10.3389/fpubh.2019.00326)
Supplement: Supplementary file 1 [file Presentation_1.pdf]

SUPPLEMENTARY MATERIAL

HIV Disease Progression among ART Patients in Zimbabwe: A Multistate Markov Model

Zvifadzo Matsena Zingoni[1, 2*], Tobias F. Chirwa[1], Jim Todd[3] and Eustasius Musenge[1]

[1]Division of Epidemiology and Biostatistics, School of Public Health, Faculty of Health Sciences, University of the Witwatersrand, Johannesburg, South Africa

[2]Ministry of Health and Child Care, National Institute of Health Research, Causeway, Harare, Zimbabwe

[3]Department of Population Health, London School of Hygiene and Tropical Medicine, London, United Kingdom

***Correspondence:**
Zvifadzo Matsena Zingoni
zmatsena28@gmail.com

Appendix A

The Chapman-Kolmogorov Example of computing probabilities

The transition intensities, $\lambda_{jk}(t)$, often termed the rates or force of transmission for infectious diseases, from state j to k is defined as

$$\lambda_{jk}(t) = \lim_{\delta t \to 0} \frac{p_{jk}(t, t+\delta t)}{\delta t} = \frac{d}{dt}\lambda_{jk}(t)\bigg|_{t=0} \tag{1}$$

where the time interval is defined by $(t, t+\delta t)$ and the transition states take the values $j = 1,2,3,4,5$ $k = 1,2,3,4,5$ and which satisfies these conditions: $\sum_{k \in S} \lambda_{jk} = 0$ for all j and the diagonal entries are defined by conversion as $\lambda_{jj}(t) = -\sum_{j \neq k} \lambda_{jk}(t)$ for all $j \in S$. The probability from state j to k is defined by p_{jk}. Once the transition intensity matrix, $P_{jk}(t)$, is known, transition probabilities can be estimated by simplifying a set of differential equation known as the Kolmogorov-Chapman differential equations defined as:

$$\frac{d}{dt}P_{jk}(t) = \sum_{\forall z} P_{jz}(t)\lambda_{zk} \quad \forall j,k \tag{2}$$

where z is the pass-through state for a transition j to k, that is $j < z < k$.

For this current study, a five-state time homogenous model was assumed of which the states were defined based on WHO clinical cut off points for ART initiation. At any given time point, the individual state is defined by four CD4 cell counts states or whether the patient is dead (fifth

state) as illustrated in Figure 1 of the main manuscript. The four CD4 states are bidirectional, meaning a patient can make a backward or forward movement from these states while the fifth state is absorbing. Also, an individual can be in any of these states making movements, or some may not make any transitions from these states; hence they remain in the same state over time.

To understand the model formulation, we assumed that between two time points $(t, t+\delta t)$, a transition occurs from state j to state k. Secondly, if a patient makes a transition with a rate of λ_{jk} where $k = j-1$ the patients would have indicated a positive movement as he/she would have moved from a bad to a good state. Practically, regarding ART uptake, such transitions can be explained by treatment efficacy; hence a patient would have shown immune recovery. This is termed backward transition, and it is a good sign for ART efficacy. Reversely, a patient can make a transition with a rate of λ_{jk} where $k = j+1$. This forward movement is alarming in ART since it is a sign of immune deterioration. This can be a result of ART defaulting, adherence issues and treatment failure in some cases. Patients would have moved to a lower CD4 cell count, and it is a cause of concern when monitoring ART patients. Not all model states are bidirectional, the transition from any of the four states (1, 2, 3, 4) to state 5 (absorbing) at a rate of λ_{jk} where $k = 5$ can occur as well . Lastly, an individual can remain in the same state over time at a rate of $\lambda_{jj} = -\lambda_j$. From these background assumptions, the transition rate matrix $(Q(t))$ for the prosed model displayed in Figure 1 can be written as:

$$Q(t) = \begin{bmatrix} -(\lambda_{12}+\lambda_{13}+\lambda_{14}+\lambda_{15}) & \lambda_{12} & \lambda_{13} & \lambda_{14} & \lambda_{15} \\ \lambda_{21} & -(\lambda_{21}+\lambda_{23}+\lambda_{24}+\lambda_{25}) & \lambda_{23} & \lambda_{24} & \lambda_{25} \\ \lambda_{31} & \lambda_{32} & -(\lambda_{31}+\lambda_{32}+\lambda_{34}+\lambda_{35}) & \lambda_{34} & \lambda_{35} \\ \lambda_{41} & \lambda_{42} & \lambda_{43} & -(\lambda_{41}+\lambda_{42}+\lambda_{43}+\lambda_{45}) & \lambda_{45} \\ 0 & 0 & 0 & 0 & 0 \end{bmatrix}$$

Once there is a complete transition intensity matrix, as shown above, the transition probabilities matrix can be estimated using the Chapman-Kolmogorov forward differential equations. These equations generally link the relationship between the transition intensities and the transition probabilities. Employing this technique, yield the following set of equations:

$$\frac{d}{dt}P_{j1}(t) = -\left(\lambda_{12}+\lambda_{13}+\lambda_{14}+\lambda_{15}\right)P_{j1}\left(t\right)+\lambda_{21}P_{j2}\left(t\right)+\lambda_{31}P_{j3}\left(t\right)+\lambda_{j4}P_{j4}\left(t\right) \quad \text{for } j=1,2,3,4 \tag{3}$$

$$\frac{d}{dt}P_{j2}(t) = \lambda_{12}P_{j1}\left(t\right)-\left(\lambda_{21}+\lambda_{23}+\lambda_{24}+\lambda_{25}\right)P_{j2}\left(t\right)+\lambda_{32}P_{j3}\left(t\right)+\lambda_{42}P_{j4}\left(t\right) \quad \text{for } j=1,2,3,4 \tag{4}$$

$$\frac{d}{dt}P_{j3}(t) = \lambda_{13}P_{j1}\left(t\right)+\lambda_{23}P_{j2}\left(t\right)-\left(\lambda_{31}+\lambda_{32}+\lambda_{34}+\lambda_{35}\right)P_{j3}\left(t\right)+\lambda_{43}P_{j4}\left(t\right) \quad \text{for } j=1,2,3,4 \tag{5}$$

$$\frac{d}{dt}P_{j4}(t) = \lambda_{14}P_{j1}\left(t\right)+\lambda_{24}P_{j2}\left(t\right)+\lambda_{34}P_{j3}\left(t\right)-\left(\lambda_{41}+\lambda_{42}+\lambda_{43}+\lambda_{45}\right)P_{j4}\left(t\right) \quad \text{for } j=1,2,3,4 \tag{6}$$

$$\frac{d}{dt}P_{j5}(t) = \lambda_{15}P_{j1}\left(t\right) + \lambda_{25}P_{j2}\left(t\right) + \lambda_{35}P_{j3}\left(t\right) + \lambda_{45}P_{j4}\left(t\right) = \sum_{z=1}^{4} P_{jz}\left(t\right)\, \lambda_{z5} \text{ for } j = 1,2,3,4 \tag{7}$$

These displayed equations (3 to 6), represent the transition probabilities and affirms the transition scenarios of bidirectional for state 1, 2, 3 and 4 while equation (7) represents the absorbing state transitions.

An example of the Chapman –Kolmogorov equation in relation to this study for a unique solution which can be recovered from the transition intensities through the product integration, to determine the probability to move from state 1 to state 5, i.e. dying from state 1 will be:

$({}_tP_x^{15})$ at a given age $\boldsymbol{x}$ will be:

$${}_{dt+t}P_x^{15} = \left({}_tP_x^{11}.{}_{dt}P_{x+t}^{15}\right) + \left({}_tP_x^{12}.{}_{dt}P_{x+t}^{25}\right) + \left({}_tP_x^{13}.{}_{dt}P_{x+t}^{35}\right) + \left({}_tP_x^{14}.{}_{dt}P_{x+t}^{45}\right) + \left({}_tP_x^{15}.{}_{dt}P_{x+t}^{55}\right) \tag{8}$$

Using $\lambda_{x+t}^{jk} = \mu_{x+t}^{jk}$ from Figure 1 shown, then ${}_{dt}P_{x+t}^{jk} = \mu_{x+t}^{jk}.dt + o(dt)$ and ${}_{dt}P_{x+t}^{55} = 1$ since a dead person stays as such, substituting these into equation (8) above gives:

$${}_{dt+t}P_x^{15} = {}_tP_x^{11}.\mu_{x+t}^{15}.dt + {}_tP_x^{12}.\mu_{x+t}^{25}.dt + {}_tP_x^{13}.\mu_{x+t}^{35}.dt + {}_tP_x^{14}.\mu_{x+t}^{45}.dt + {}_tP_x^{15}.1 + o(dt) \tag{9}$$

Taking the ${}_tP_x^{15}$ term and dividing through by dt and then taking limits of equation (9) as $dt \to 0$ using $\lim_{dt \to 0} \frac{0(dt)}{dt} = 0$, we get the expression below:

$$\lim_{dt \to 0}\left(\frac{{}_{dt+t}P_x^{15} - {}_tP_x^{15}}{dt}\right) = \frac{\partial}{\partial t}\left({}_tP_x^{15}\right)$$
$$= {}_tP_x^{11}.\mu_{x+t}^{15} + {}_tP_x^{12}.\mu_{x+t}^{25} + {}_tP_x^{13}.\mu_{x+t}^{35} + {}_tP_x^{14}.\mu_{x+t}^{45} \tag{10}$$

Therefore,

$${}_tP_x^{15} = \int {}_tP_x^{11}.\mu_{x+t}^{15} + {}_tP_x^{12}.\mu_{x+t}^{25} + {}_tP_x^{13}.\mu_{x+t}^{35} + {}_tP_x^{14}.\mu_{x+t}^{45}\, dt \tag{11}$$

can be solved by a separate method and integration factor method and can be simplified in Bayesian WinBUGS software. However, using some properties of occupancy and that exit from a state is the negation of that, this expression can be shown to be:

$${}_tP_x^{15} = \frac{e^{-\int_0^t\left(\sum_{k=2}^{5}\mu_{x+t}^{1k}\right)dt}\left(\mu_{x+t}^{15}\right)}{\sum_{k=2}^{5}\mu_{x+t}^{1k}} + \frac{1-e^{-\int_0^t\left(\sum_{k\neq 2}^{5}\mu_{x+t}^{2k}\right)dt}\left(\mu_{x+t}^{25}\right)}{\sum_{k\neq 2}^{5}\mu_{x+t}^{2k}} + \frac{1-e^{-\int_0^t\left(\sum_{k\neq 3}^{5}\mu_{x+t}^{3k}\right)dt}\left(\mu_{x+t}^{35}\right)}{\sum_{k\neq 3}^{5}\mu_{x+t}^{3k}} + \frac{1-e^{-\int_0^t\left(\sum_{k\neq 4}^{5}\mu_{x+t}^{4k}\right)dt}\left(\mu_{x+t}^{45}\right)}{\sum_{k\neq 4}^{5}\mu_{x+t}^{4k}} + C \tag{12}$$
